# Nebraska Veterinary Medical Association

**Published:** 1891-04

**Authors:** S. E. Cosford

**Affiliations:** Secretary


*Nebraska Veterinary Medical Association.*—The semi-annual meeting of the Nebraska Veterinary Medical Association was held March 10, 1891, in the parlors of the Merchant's Hotel, Omaha, Neb. The meeting was called to order by the President, Dr. E. Noble, and the following members reported to roll-call: Drs. Blackwell, Cosford, Ebbitt, Lord, Noble, Ramacciotti, and Young. Guest, Dr. S. Stewart, of Council Bluffs, Ia. Dr. Wilson, of Lincoln, was elected to membership. Dr. Lord presented a paper on Antiseptic Surgery, which was well received and thoroughly discussed. Dr. Young's paper was postponed by request. Dr. Stewart presented a paper on the use of Cannabis Indica in colic, which also elicited liberal discussion. The essayists were tendered a vote of thanks. On motion Dr. Stewart was elected an honorary member of the association.

The president appointed Drs. Young, Taylor and Dickey essayists for the next meeting, which will be held at Lincoln next September.

A motion prevailed to create a legislative committee, to secure if possible, the passage of an Act to regulate the practice of Veterinary Medicine and Surgery, now pending in the Legislature. The president appointed as such committee Drs. Ramacciotti, Lord and Cosford.

After the disposal of routine business the meeting adjourned.

S. E. COSFORD, *Secretary.*

